# Evolutionary history of *Podarcis tiliguerta* on Corsica and Sardinia

**DOI:** 10.1186/s12862-016-0860-4

**Published:** 2017-01-19

**Authors:** V. Rodríguez, J. M. Buades, R. P. Brown, B. Terrasa, V. Pérez-Mellado, C. Corti, M. Delaugerre, J. A. Castro, A. Picornell, M. M. Ramon

**Affiliations:** 1 Laboratori de Genètica, Universitat de les Illes Balears, Palma de Mallorca, Spain; 2 School of Natural Sciences & Psychology, Liverpool John Moores University, Liverpool, UK; 3 Departamento de Zoología, Universidad de Salamanca, Salamanca, Spain; 4 Museo di Storia Naturale de ll’Università di Firenze, Sezione di Zoologia “La Specola”, Firenze, Italy; 5 Conservatoire du Littoral, Bastia, France

**Keywords:** *Podarcis tiliguerta*, *Lacertidae*, Corsica and Sardinia Islands, Mitochondrial DNA, Nuclear DNA, Species tree

## Abstract

**Background:**

*Podarcis tiliguerta* is a wall lizard endemic to the Mediterranean islands of Corsica and Sardinia. Previous findings of high mtDNA and morphological diversity have led to the suggestion that it may represent a species complex. Here, we analysed mitochondrial and nuclear markers (mtDNA, 3110 bp; 6 nDNA loci, 3961 bp) in *P. tiliguerta* sampled from thirty-two localities across Corsica and Sardinia.

**Results:**

We find much greater intraspecific genetic divergence than between sister species of other Mediterranean island *Podarcis*, i.e., between *P. lilfordi* and *P. pityusensis*. We detected three mtDNA clusters in Corsica (North, South-East and South-West) and either two or three in Sardinia (North vs. South) depending on the clustering method. Only one or two nDNA groups were identified within each main island (again, depending on the method). A Bayesian time-calibrated multispecies coalescent tree was obtained from mtDNA and provided statistical support for a Miocene origin of the species (13.87 Ma, 95% HPD: 18.30–10.77 Ma). The posterior mean divergence time for the Corsican and Sardinian lineages was 12.75 Ma ago (95% HPD: 16.94–9.04 Ma).

**Conclusion:**

The results support the evolutionary distinctiveness of Corsican and Sardinian populations and also indicate a lack of post-divergence migration despite periods of contact being possible. Further to this, species delimitation analyses of Corsican and Sardinian lineages provided statistical support for their recognition as distinct (sister) taxa. Our results provide new insights into the biogeography of the Mediterranean biodiversity hotspot, and contribute important findings relevant to the systematics and evolution of this speciose lizard genus.

**Electronic supplementary material:**

The online version of this article (doi:10.1186/s12862-016-0860-4) contains supplementary material, which is available to authorized users.

## Background

Islands provide many of the world’s biodiversity hotspots. They continuously generate new species with the term “speciation machines” having been coined to describe this phenomenon [1]. These high speciation rates are facilitated by specific island characteristics that lead to a wealth of ecological, biogeographic and evolutionary processes. These include: natural fragmentation, long term isolation, high altitudes creating habitat heterogeneity, complex intra-island landscapes, and island emergence due to volcanism and eustatic sea-level changes.

The Mediterranean basin was one of the first 25 Global Biodiversity Hotspots to be named [2] and is characterized by substantial island diversity [3]. The Tyrrhenian region within the Mediterranean is particularly diverse due to the influence of complex paleogeographical and historical factors [4, 5]. It includes Sardinia and Corsica, two of the largest and highest (1834 m and 2710 m, respectively), Mediterranean Islands, which are separated by a strait of only 11 km. They contain high levels of endemism including several hundred endemic plants. Geological history has undoubtedly played a role in generating this diversity. A land bridge maintained the connection between the islands and Europe [6] until their separation, completed 9 Ma ago [7, 8]. This was subsequently lost although connections between the Corsica–Sardinia archipelago and Europe and North Africa were re-established through desiccation of the Mediterranean basin during the Messinian Salinity Crisis (MSC; 5.96–5.33 Ma) [9, 10]. Corsica and Sardinia have since been temporarily connected as a result of more minor changes in sea level during glacial periods in the Pleistocene, with the last contact being during the Last Glacial Maximum (LGM) [11].

The lizard genus *Podarcis* encompasses about 19 species and is widespread across the Mediterranean. *Podarcis tiliguerta* is endemic to Corsica and Sardinia. It exhibits a great intraspecific variability in morphological traits, especially colour pattern and melanism, the latter found within populations from small islands away from the main islands [12–17]. This variation has led to the description of several microinsular subspecies [18, 19]. Morphological [20, 21] and genetic studies, based on both allozymes [22] and mitochondrial sequences [23–25], have evidenced the existence of two or possibly three divergent genetic clusters within the Corsica-Sardinia archipelago. The underlying reasons for this divergence remain unexplained, but its magnitude has led some authors to suggest that *P. tiliguerta* might represent an insular species complex [20, 24]. Similar findings have been documented for other *Podarcis* from much wider geographic areas, e.g., *P. hispanica*, from the Iberian peninsula [26] and *P. erhardii*, from Greece [27]. Objective coalescent-based statistical analyses of species delimitation may provide an important step towards robust assignment of new taxonomic units based on genetic groups within the putative complex.

This study aims to provide the most detailed analysis of genetic variation of *P. tiliguerta* to date, using both mitochondrial (mtDNA) and nuclear DNA (nDNA) markers. We use these data to reveal new insights into the patterns and causes of the genetic diversity. We examine two important aspects of the evolutionary history of *P. tiliguerta*, namely the number of lineages and timing of divergence between these lineages and from other *Podarcis*. In addition, we investigate the historical biogeography of *P. tiliguerta* in relation to known physical changes in the region.

## Methods

### Sampling

**Fig. 1:**
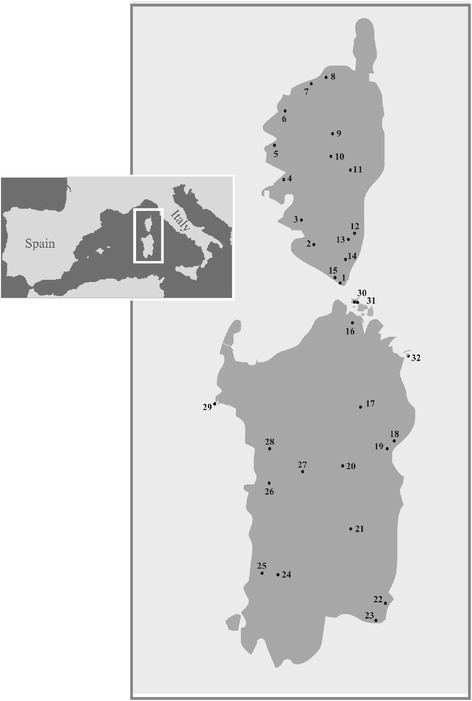
Sites at which *Podarcis tiliguerta* were sampled in Corsica, Sardinia and adjacent islands

Forty-one *Podarcis tiliguerta* were captured under license from 15 localities in Corsica, 13 localities in Sardinia (in May 2011) and four offshore islands and islets (September 2012) by noosing, with the aim of covering the main geographical regions (Fig. 1 and Table 1). Tail-tips were removed and stored in 100% ethanol before releasing individuals at sites of capture.

**Table 1:** Localities and *P. tiliguerta* specimens sampled

	Localities	Samples
	Corsica Island	
1	Bonifacio	TCO1/17
2	Sartène	TCO2
3	Filitosa	TCO3
4	Sant Bastiano (Calcatoggio)	TCO4
5	Les Chalanches (Piana)	TCO5
6	Casa Vecchia (Calvi)	TCO6
7	L’Île Rousse	TCO7
8	Pietra Moneta	TCO8
9	Corte	TCO9/18
10	Vivario	TCO10
11	Ghisonaccia	TCO11/19
12	Ribba (San Cavino Di Carbini)	TCO12
13	Casteddu d’Araghju (Lecci)	TCO13
14	Chera (Sotta)	TCO14
15	La Trinité (Bonifacio)	TCO20/21
	Sardinia Island	
16	Gallura	TSA3
17	Romanzesu (Bitti Nuoro)	TSA1
18	Dorgali (Nuoro)	TSA11
19	Su Gologone (Oliena Nuoro)	TSA10
20	Fonni	TSA5
21	Flumendosa lake (Cagliari)	TSA13
22	Costa Rei (Cagliari)	TSA19
23	Villasimius (Cagliari)	TSA12/18
24	Locanda dil Parco (foresta demaniale di Montimannu, Villacidro)	TSA15
25	Fluminimaggiore	TSA20
26	Narbolia (Oristano)	TSA17
27	Nuraghe Sorradile (Oristano)	TSA2
28	Nuraghe Nuradeo	TSA4
	Sardinian islands and islets	
29	Foradada Island (Alghero Sassari)	Tf1–3
30	Paduleddi islet (La Maddalena archipelago)	Tp1–3
31	Stramanari islet (La Maddalena archipelago)	Ts1
32	Molara Island	TSA8

### DNA Amplification and sequencing

A standard phenol-chloroform protocol was used for DNA extraction [28]. Six non-overlapping mtDNA fragments were amplified and sequenced for each specimen containing sequences from the following genes: i) partial 12S rRNA, ii) partial tRNA_Glu_ and partial cytochrome b (cytb), iii) partial cytb and partial tRNA_Thr_, iv) partial control region (CR), v) two partial subunits of the NADH dehydrogenase gene and associated tRNAs (referred to as ND1, ND2, tRNA_Ile_, tRNA_Gln_, and tRNA_Met_) and vi) partial 16S rRNA. Primers and amplification conditions are the same as those used in our previous studies of *Podarcis* [29–31] (see Additional file 1), with the exception of the light strand primer (L14143; see Additional file 1) for amplification of fragment (ii) (above) in the Sardinian specimens. The 16S rRNA primers were obtained from Carranza et al. [32].

Six partial nuclear genes were sequenced: i) melanocortin-1 receptor gene (*MC1R*), ii) recombination-activating gene 1 (*RAG1*), iii) apolipoprotein B gene *APOBE28*, iv) lipoprotein gene *BLC9L*, v) transcription factor gene *KIAA2018* and vi) kinesin family member *KIF24*. Primers [33–35] are described in Additional file 1.

Sequencing of both strands of PCR products was carried out on an automated ABI 3130 sequencer (Applied Biosystems, Foster City, CA, USA) using a BigDye® Terminator v. 3.1 Cycle sequencing kit (Applied Biosystems, Foster City, CA, USA), and edited and aligned using BioEdit v.7.0.5.2. [36].

### Analyses of genetic variability and structuring

DnaSP v.5.10.01 [37] was used to obtain basic genetic diversity indices, test for neutrality and phase nuclear data. Potential recombination events were assessed using the Pairwise Homoplasy Index (phi) test [38] implemented in SplitsTree v. 4 [39].

Relationships between nuclear DNA haplotypes were explored using statistical parsimony networks, with a 95% connection limit (program: TCS v.1.21 [40]).

Analysis of molecular variance (AMOVA) (program: Arlequin v.3.1.1 [41]) was used to examine genetic structuring between primary geographic groups (Corsica vs. Sardinia and Sardinian islands).

Bayesian phylogeographical and ecological clustering (BPEC) [42, 43] was used to identify genetically distinct geographical population clusters in Corsica and Sardinia. BPEC relies on parsimony in order to reduce the number of candidate trees. Different haplotype trees and migration events are explored through the Markov chain Monte Carlo (MCMC) sampler. Migration events are assumed to occur when a haplotype (with or without a mutation from its parent haplotype) migrates to a new geographical cluster. Locations were specified as latitudes and longitudes recorded during fieldwork using a motion X-GPS HD V21.0 Build 1737R (Fullpower Technologies Inc.). For each run, a strict parsimony criterion was applied and the maximum number of migrations was 4. The MCMC chain was run for 10 million iterations with 20,000 steps saved. Separate analyses were performed on Corsica and Sardinia. A subsample of 1371 mtDNA sites were selected for this analysis due to high levels of mtDNA divergence (saturation of third codon positions and other regions were detected using an appropriate test [44, 45]). The analysed sequence comprised: 12S rRNA (359 bp), CR (465 bp) and cytb codon positions 1 and 2 (548 bp). The geographical distributions of alleles were similarly analyzed for each nuclear locus, independently.

For comparison, an alternative genetic clustering procedure implemented within the program BAPS v.5.3 [46] was used to infer population structure without reference to geographical location. It was applied to all nuclear markers together, and to mtDNA alone. The number of clusters was determined and admixture analyses were executed to infer the ancestral source of the samples (without priors on geographic location).

### Species phylogeny, divergence times and species delimitation

The multispecies coalescent approach implemented in *BEAST v. 1.7.4 [47, 48]; was used to estimate divergence times and phylogenetic relationship between specific mitochondrial lineages from the main islands, Corsica and Sardinia (with surrounding islets). The method allows estimation of divergence times while taking into account ancestral polymorphism. However, it often suffers from problems associated with prior specifications [49] and achieving good mixing of the MCMC chain (particularly when multiple loci are analysed). Also, the inclusion of several poorly informative nuclear markers could have a substantial and potentially undesirable impact on the estimated divergence times [50]. For these reasons, we included only mtDNA in the analysis.

Eight partitions were used to account for heterogeneity in sequence evolution and suitable models for each partition were identified using jModeltest v.0.1.1 [51] (AICc criterion, i.e., Akaike Information Criterion corrected for small samples). We partitioned the sequence by grouping functionally similar sites from specific regions as they should have similar properties (rates in particular). These were: i) 12S rRNA, ii) CR, iii) all tRNAs, iv) 1^st^ and 2^nd^ codon positions of the ND1/ND2 sequences, v) 3^rd^ codon position of ND1/ND2, vi) 1^st^ codon position of cytb, vii) 2^nd^ codon position of cytb, viii) 3^rd^ codon position of cytb, When the selected model was not available in *BEAST, we used the most similar available model.

We included mtDNA from all *P. tiliguerta* samples together with homologous sequences from two species that comprise a sister lineage to *P. tiliguerta*: *P. lilfordi* and *P. pityusensis* [52]. Inclusion of these taxa allowed incorporation of a time-calibrated node in the analysis [31, 53] (see Additional file 2 for GenBank accession numbers). The age of the (*P. lilfordi*, *P. pityusensis*) node on the population was constrained using the normal distribution N (5.325, 0.0001), where 1 unit = 1 Ma. The MSC is a well-known physical event in the Mediterranean and ended with very rapidly rising sea levels after the opening of the Mediterranean-Atlantic connection. Some studies have suggested that refilling of the basin may have only taken only a few thousand years [9, 10, 54]. This would have separated the Gymnesic and Pityusic Islands. Evidence from other studies (e.g., [55]) indicates the suitability of narrow time constraints on (Gymnesic, Pityusic) nodes. Refilling has been dated at 5.33 Ma [9] and given the deep intermediate channel between the island groups and the evidence of a very rapid refilling of the basin, the narrow normal distribution around a mean of 5.325 Ma would appear suitable.

A strict clock model was used in *BEAST (see [56]), and the tree prior was specified by a Yule process. The MCMC chain was run for 300 million generations sampled every 10,000 steps. Tracer v. 1.5 [57] allowed visual inspection of marginal posteriors. TreeAnnotator (BEAST package) was used to combine and analyse the posterior tree samples at stationarity (trees were combined using the maximum sum of clade credibilities criterion).

Species delimitation was examined using three different approaches. First the Bayesian Phylogenetics and Phylogeography program (BPP, v. 3.1) [58, 59] was used to obtain posterior probabilities on specified nodes. BPP implements a reversible jump Markov chain Monte Carlo (rjMCMC) to search the parameter space that includes θpopulation divergence and gene trees. Analyses were performed with i) mtDNA, ii) nDNA and iii) all markers, to determine the impact of mtDNA polymorphism on species delimitation. Gamma distributions (G) with the following shape and different scale parameters were used to specify the population size, θ_s_: G(1,10) τ0 G (1,10), θ_s_ G(1,10) τ0 G(1, 2000), θ_s_ G(1, 2000) τ0 G (1,10), θ_s_ G(1, 2000) τ0 G (1, 2000). The MCMC chain was run for 400,000 steps (following a burnin of 20,000 steps), sampled every 20 steps. Each analysis was run three times to confirm consistency between runs.

Two additional approaches of species delimitation analysis suitable for single locus data (only mtDNA) were also tested. The Poisson Tree Processes model (PTP) [60] uses non-ultrametric phylogenetic trees and directly incorporates the number of nucleotide substitution in the model of speciation using a Poisson distribution to describe the probability that a substitution gives rise to a speciation event. This method delimits species under the assumption that the number of substitutions between species is considerably higher than within species. The branch lengths of the input tree are supposed to be generated by two independent classes of Poisson events, one corresponding to speciation and the other to coalescence. A bPTP analysis (a recent version of the PTP that implements Bayesian support values to the delimited species) was conducted on the bPTP web server (http://species.h-its.org/ptp/). A phylogenetic tree inferred by IQ-Tree [61] was used as input. The chain was run for 150,000 MCMC generations, 10% of samples were discarded as burn-in and sampling interval was 100.

Finally GMYC [62, 63], based on time-calibrated ultrametric phylogenetic trees and using differences in branching rates to infer species was applied. The input tree was generated in BEAST v1.8.2. [48] and the specifications were the same as the ones described above for the *BEAST analysis (8 partitions, models, strict clock, time-calibration and tree prior/yule process). Independent MCMC runs of 76 million steps were sampled every 10,000 steps (10% of samples were removed as burn-in). The GMYC analysis was conducted using this consensus tree and the single-threshold method within the SPLITS package [64] on R v 3.3.2 [65].

## Results

### Mitochondrial DNA

A total of 3110 bp of mtDNA sequence was obtained (12S rRNA 392 bp, control region 497 bp, ND1 85 bp, ND2 510 bp, cyt b 929 bp (two fragments), tRNAs 256 bp, 16S rRNA 431 bp). Sequences have been deposited in GenBank (for accession numbers see Additional file 3). The concatenated sequence contained 646 polymorphic sites, giving rise to 37 different haplotypes, which differed by a mean pairwise difference of 216.09 (±92.03). Indices of mtDNA diversity (Table 2; analyses without 16S rRNA sequences) were higher in *P. tiliguerta* than corresponding values for related species [29, 31], while non-significant neutrality tests [66–69] supported neutral evolution.

**Table 2:** Diversity estimates for the concatenated mtDNA fragment in *P. tiliguerta* (without 16S rRNA sequences) compared with others species of the Western Islands Group (*P. lilfordi*, *P. pityusensis* and *P. filfolensis*). Parameters for Corsica and Sardinia *P. tiliguerta* were also estimated independently

Species	N°	V	N° hap	Nucleotide diversity	K	Fu´s Fs (1997)	Fu and Li’s D (1993) [67]	Fu and Li’s F (1993) [67]	Fay and Wu’s H (2000) [68]	Tajima’s D (1989) [69]
*P. lilfordi*	117	189	62	0.019 ± 0.005	45.067	-1.129 ^ns^	0.652^ns^	0.860^ns^	-17.572 ^ns^	0.770^ns^
^a^(2382 bp)										
*P. pityusensis*	73	90	60	0.004 ± 0.001	9.083	-56.983***	-2.432**	-2.589**	-25.341**	-1.745*
^b^(2430 bp)										
*P. filfolensis*	31	41	20	0.004 ± 0.001	9.355	-4.097*	-0.681 ^ns^	-0.668 ^ns^	-12.409**	-0.325 ^ns^
^c^(2533 bp)										
*P. tiliguerta*	41	595	37	0.074 ± 0.003	197.126	0.840 ^ns^	1.419 ^ns^	1.425 ^ns^	-12.209 ^ns^	0.792 ^ns^
(2681 bp)										
Corsica	19	394	19	0.046 ± 0.006	122.07	-1.030 ^ns^	0.858 ^ns^	0.671 ^ns^	-77.263 ^ns^	-0.043 ^ns^
Sardinia	22	386	18	0.051 ± 0.009	137.36	4.241 ^ns^	1.510 ^ns^	1.569 ^ns^	-52.468 ^ns^	0.859 ^ns^

N°, number of individuals sampled; V, variable position; N° hap, number of haplotypes; K, average number of pairwise differences;

^ns^not significant; *P < 0.1; ** *P* < 0.05; ***P < 0.001

^a,b,c^obtained from data in Terrasa et al*.* 2009 [29]^a^, Rodríguez et al. 2013 [31]^b^ and Rodríguez et al. 2014 [30]^c^

### Nuclear data

The nuclear loci analysed were: *MC1R* (720 bp), *RAG1* (939 bp), *APOBE28* (489 bp), *BLC9L* (627–636 bp), *KIAA2018* (644–659 bp) and *KIF24* (497–518 bp). Sequences have been deposited in GenBank (Additional file 3). Basic genetic diversity indices and neutrality statistical tests [66, 69] are indicated in Table 3. The phi test [38] showed no statistically significant evidence for recombination in any gene: *MC1R* (*p* = 0.164), *APOBE28* (*p* = 0.369), *KIAA2018* (*p* = 0.095), *BLC9L* (*p* = 0.467), *KIF24* (*p* = 0.322), *RAG1* (*p* = 0.193).

**Table 3:** Diversity estimates for six nuclear exons in *P. tiliguerta* (length polymorphisms are included in these analyses)

Exon gene	Length (bp)	N° ind.	V	N° hap	Nucleotide diversity	K	Fu’s Fs (1997) [66]	Tajima D (1989) [69]
*MC1R*	720	39	47	56	0.006 ± 0.0003	4.076	- 0.133^ns^	- 0.064^ns^
*RAG1*	939	38	70	52	0.015 ± 0.0004	6.045	- 0.424^ns^	- 0.086^ns^
*APOBE28*	489	37	47	41	0.006 ± 0.0006	2.961	- 0.367^ns^	- 0.055^ns^
*BLC9L*	636	38	67	56	0.009 ± 0.0006	5.544	- 0.139^ns^	- 0.052^ns^
*KIAA2018*	659	39	71	66	0.020 ± 0.0006	13.000	- 0.262^ns^	- 0.039^ns^
*KIF24*	518	34	75	55	0.021 ± 0.0008	10.449	- 0.491^ns^	- 0.078^ns^
Concatenated								
Corsica (*n* = 38)	3961		225	38	0.010 ± 0.006	37.516	0.288^ns^	- 0.083^ns^
Sardinia (*n* = 40)	3961		244	39	0.010 ± 0.003	38.063	0.353^ns^	- 0.076^ns^
* P. tiliguerta* (*n* = 78)	3961		377	77	0.011 ± 0.002	41.985	0.360^ns^	- 0.061^ns^

N°, number of individuals; V, number of variable position; N° hap, number of haplotypes; K, average number of pairwise differences;

n, number of sequences resulted of phased data, ^ns^ not significant

Length polymorphisms were detected in three nuclear genes, including STRs (short tandem repeats) and INDELs (insertion-deletions). We briefly describe them here. The *KIAA2018* sequence contained a polymorphic microsatellite with a proline repeat (CCT) ranging from 4-9. The (CCT)_7_ repeat was the most frequent (see Additional file 4). Only three individuals showed an amino acid change within this repeat. These were: i) two Sardinian samples TSA8 (CTT) and TSA18 (CYT) showed a leucine substitution in the fifth repeat, and ii) the Corsica sample TCO9 showed eight repeats with a threonine substitution in the final repeat. An STR was also observed within the *BLC9L* sequence with a variable number of repeats for glutamine and proline. The common structure was (Gln)_3-4_ (Pro)_5-7_ (Glu)_5_ with the unique exception of a Corsican sample (TCO2) that showed an histidine: (Gln)_4_ (Pro)_5_ (His) (Gln)_5_. Three different INDELs were detected in *KIF24* sequences: i) a 9 bp deletion (CATTTTGGT) in specimen TSA5 and ii) a 12 bp insertion (AAGGACTTTGGG) in TSA1–3, TSA5, TSA13 and Tf1–2, iii) 6 bp deletions (GAAAGC) in TCO6 and TCO10; and (AGAAAG) in TCO9. Note that i) and ii) occurred only in Sardinia, while iii) occurred only in Corsica.

### Genetic structure of the insular populations

**Fig. 2:**
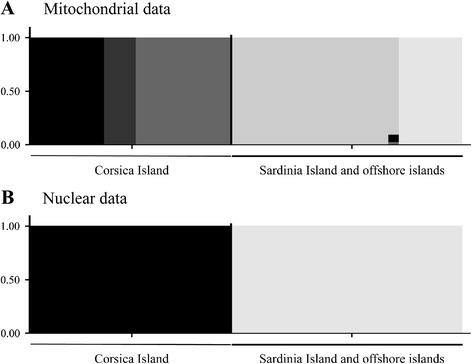
Overall genetic structure inferred from **a**) mtDNA and **b**) concatenated nuclear loci across the two main geographic regions used in this study. Mixture analyses were estimated by BAPS software. In the bar plot, vertical bars represent individuals and proportions of admixture, with colours corresponding to ancestral sources

The mtDNA variance was partitioned as 51.01% (i.e., F_ST_ = 0.51) between Corsica and Sardinia (and offshore islands) and the remaining variance within these regions (AMOVA analysis). BAPS analysis of the mtDNA locus defined five genetic clusters (lnL = -8303.607) (Fig. 2a). The analysis distinguishes three Corsican groups: i) cluster A includes lizards from the south-western region (TCO2–4), ii) cluster B includes lizards from the south-east (TCO1, 12–14, 17, 20–21) and iii) cluster C includes lizards from the north of Corsica (TCO5–11, 18–19). Two Sardinian clusters are identified: cluster D includes specimens corresponding to localities in the south of the island (TSA12–13, 15, 18–20) and cluster E contains *P. tiliguerta* from Sardinian islands and islets (Molara Island, Foradada Island, Paduleddi Islet and Stramanari Islet) and from the north of the main island (TSA1–5, 10–11, 17).

**Fig. 3:**
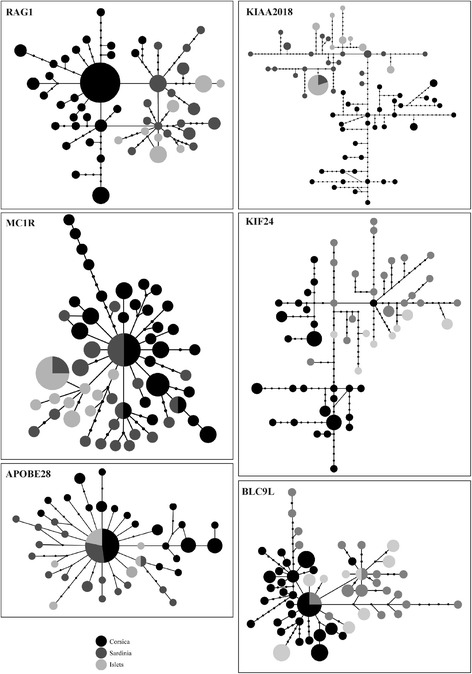
Nuclear haplotype networks for *Podarcis tiliguerta*

AMOVA of the nuclear loci indicated that 18.2% (i.e., F_ST_ = 0.182) of the variance occurred between Corsica and Sardinia (and offshore islands) with most of the variance being within these regions. Only two genetic clusters were defined by a BAPS analysis of concatenated nDNA (lnL = -5640.911). The first included all Corsican individuals, and the second included Sardinian and individuals from small islet populations (Fig. 2b). The TCS analysis indicated that two loci (*APOBE28* and *MC1R*) show star-like parsimony networks, with a main haplotype shared by Corsican and Sardinian samples, and derived haplotypes belonging to both islands (Fig. 3). In contrast, *RAG1* and *BLC9L* show compound star-like networks consisting of two central haplotypes, corresponding to Corsica and Sardinia, separated by one mutational step. Finally, the two other networks (*KIF24* and *KIAA2018*) exhibit a clear separation between Corsican and Sardinian samples, together with a much higher differentiation between haplotypes (30 mutational steps). The *KIAA2018* sequences show greatest diversity of all nuclear loci analysed, characterised by 66 different haplotypes.

**Fig. 4:**
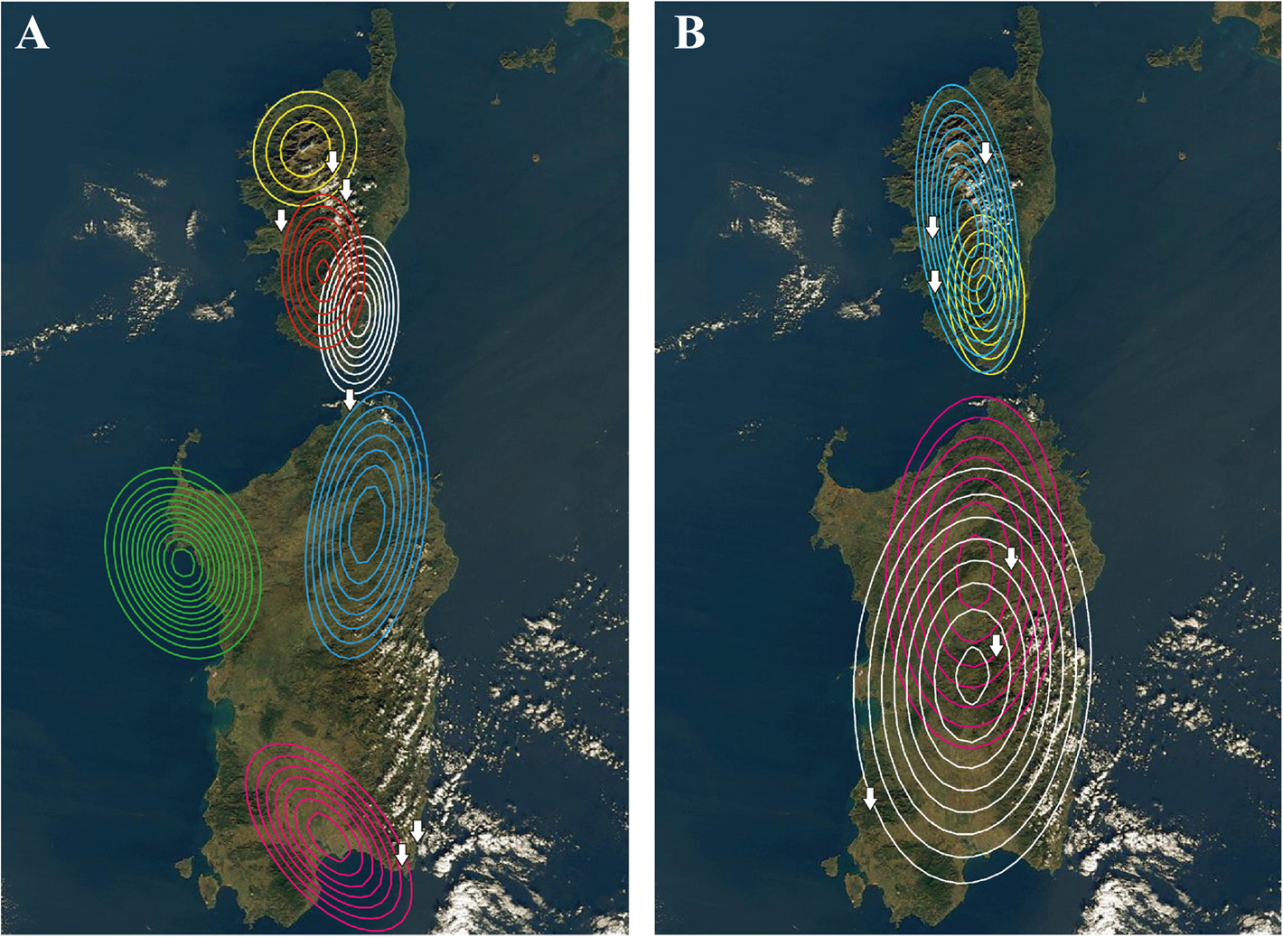
Analyses of Bayesian phylogeographical and ecological clustering (BPEC): **a**) using mtDNA or **b**) RAG1 as example of nuclear DNA. Each coloured contour plot indicates a different phylogeographical clusters. The contour plots are centred at the ‘centre’ of each population cluster, and the coloured regions show the radius of 50% concentration contours around it. *White arrows* show the ancestral locations with the highest posterior probability for each island

Mitochondrial haplotypes from Sardinia were assigned by BPEC to three phylogeographic clusters with high posterior probabilities (>0.7) (Fig. 4a), with most likely ancestral locations being Gallura, Villasimius and Costa rei (see Fig. 1 and Table 1). Three clusters were also obtained for Corsica (Fig. 4a) with likely ancestral locations being Sant Bastiano, Corte and Vivario (see Fig. 1 and Table 1). Low posterior cluster assignment probabilities were obtained for eight Corsican haplotypes (~0.4–0.5), and one Sardinian (~0.5) haplotype. Geographical structuring in Corsica corresponded to the north, south-east and south-west of the island, while in Sardinia three geographical groupings were detected: i) north (with some offshore islands), ii) south and iii) Foradada Island (Fig. 4a). BPEC analyses of nuclear loci assigned haplotypes to four phylogeographic clusters with generally high posterior probabilities: two of them, in Corsica and two, in Sardinia (Fig. 4b). However all nuclear genes divide the main islands into northern and southern regions.

### Species phylogeny, divergence times and species delimitation

Preliminary gene tree analyses were performed on all individual loci prior to the Bayesian multispecies coalescent analysis. These analyses ruled out the possibility that *P. tiliguerta* was polyphyletic relative to the other closely related taxa that were included (*P. hipanica, P. guadarramae, P. virescens, P. liolepis, P. lilfordi*, *P. pityusensis*, and *P. filfolensis*) (data not shown).

**Fig. 5:**
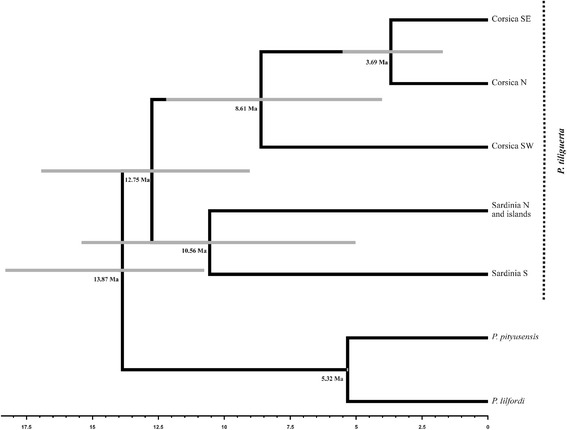
Mitochondrial populations tree chronogram estimated by *BEAST in *P. tiliguerta. P. lilfordi* and *P. pityusensis* are used as outgroups. *Grey* bars correspond to the 95% highest posterior density intervals for each divergence time

The species tree (Fig. 5), based on mtDNA, provided a posterior mean for the divergence of *P. tiliguerta* from (*P. lilfordi*, *P. pityusensis*) at 13.87 Ma (95% highest posterior density, HPD: 18.30–10.77 Ma), and that for the divergence of Corsica and Sardinia lineages, at 12.75 Ma (95% HPD: 16.94–9.04).

All BPP analyses supported the two species (Corsica, Sardinia) model (P = 1.00) irrespective of the prior specifications for θ_s_ and τ0 and whether mtDNA was included or excluded from the analyses.

The trees obtained from the bPTP analysis (PTP_ML and PTP-sh) (excluding outgroups) showed 11 groups with high posterior probabilities (≥0.91). Of the 11 clusters, 8 comprised only 1 sample. The GMYC approach detected 9 clusters within *P. tiliguerta* with a total of 19 entities, excluding outgroups and with posterior probabilities ≥ 0.85. The different clusters detected by these two analyses corresponded to different clades detected within Corsica and Sardinian islands, but none of these groups comprised both Corsica and Sardinia individuals, further supporting species delimitation of these two island forms. Additional information about PTP and GMYC analyses is available on request.

## Discussion

The most striking finding is the high degree of genetic variability detected within the taxon *Podarcis tiliguerta*. This has already been suggested by two previous studies that analysed protein mobility and mtDNA [22, 24]. Other insular Mediterranean species, such as *P. pityusensis*, *P. lilfordi* or *P. filfolensis* show lower levels of divergence, although they inhabit smaller islands*.* For exemple, the mean pairwise differences for comparable mtDNA regions is more than four times higher than in *P. lilfordi*, and is almost 22 times greater than in *P. filfolensis* or *P. pityusensis.* In addition, intraspecific nDNA variability also appears to be greater *P. tiliguerta* than in other *Podarcis* which confirms the extreme genetic diversity observed within this species [24, 70].

The high diversity is primarily accounted for by strong geographical structuring with a deep, ancient divergence between Corsican and Sardinia lineages as well as substantial within-island divergence. Early analyses of allozymes and short mtDNA fragments indicated two genetic groupings: Corsica and Sardinia [22, 24]. Subsequent analyses, with wider sampling, revealed three mtDNA groups: two in Sardinia and one in Corsica [25, 70]. Here, we detect three geographically-structured mtDNA groups within Corsica, corresponding to the north, south-east and south-west of the island. We also detect two/three (depending on the analysis) spatial groupings of mtDNA within Sardinia: one of which corresponds to the north of the main island and associated smaller islands, while the other corresponds to the south.

As expected, the nuclear data detected fewer clusters. The BAPS analyses revealed two groups: Corsica and Sardinia, while the slightly more sensitive BPEC analyses revealed two additional clusters within each island. Bruschi et al*.* [20] studied *P. tiliguerta* using morphological characters and suggested hybridization was occurring in southern Corsican populations due to introductions of individuals from Sardinia which might contribute to these patterns. However, our nuclear and mtDNA analyses found no evidence of this. Although some nuclear alleles were shared between Corsican and Sardinian populations, the lack of any such patterns in the mtDNA suggests that incomplete lineage sorting is the most likely cause.

The ancestral species node for *P. tiliguerta* was dated at 12.75 Ma (Fig. 5; 95% HPD: 16.94–9.04 Ma) confirming preliminary divergence time estimates (13 Ma) [71]. *P. pityusensis* and *P. lilfordi* are related species to *P. tiliguerta*, including all them in the named “Western Islands group” [72]. This clearly predates the intraspecific divergence within: i) *P. lilfordi* which dates to the early Pleistocene [53], ii) *P. filfolensis* which also diverged in the Pleistocene [30] and iii) *P. pityusensis* which diverged in the mid-late Pleistocene [31]. The divergence time between Corsica and Sardinia far exceeds the usual limits for distinct species within the Squamata [73, 74]. Reciprocal monophylia between the islands was found for several loci, which supports our detection of species delimitation (although not for all loci, as would be expected between distinct species). This, and the general concordance with and among nuclear markers, explains why our species delimitation analysis provides such strong support for separation of *P. tiliguerta* into two species. In a future study we intend to provide formal (morphological and ecological) descriptions of these new taxa.

*P. tiliguerta* appears to have originated by allopatric speciation, from continental ancestors that colonized the Corsica-Sardinia microplate. According to Arnold et al. [52], the Lacertini spread and diversified around 16–12 Ma BP producing the ancestors of the present genera, so genus origin postdates the isolation of the Corsica-Sardinia microplate from the Iberian plate (29 Ma). This indicates that the ancestors reached it subsequently via the land bridge that it maintained with the continent during its rotation. Later, the microplate split into two islands (15–9 Ma ago [7, 8]) leading to isolation of the Sardinian and Corsican lineages, concordant with our divergence time estimate. Since separation, the heterogeneous orography of Corsica and Sardinia could have played an important role in determining intraspecific diversity.

## Conclusions

In summary, we analysed mtDNA and nuclear markers in *P. tiliguerta* and found high genetic variability and a deep genetic structure, corresponding to deep Miocene divergence between Corsica and Sardinia. In addition, we detected substantial within-island divergence but were unable to relate it to any geological or other events in the islands past. This study will motivate and contribute evidence to a taxonomic reassessment of *P. tiliguerta* with respect to the Corsican and Sardinian forms.

## Additional files

Additional file 1: Primers sequences used in amplification and sequencing. (DOCX 17 kb)
Additional file 2:
*Podarcis spp* analysed as outgroup in *BEAST analyses. (DOCX 11 kb)
Additional file 3: Sample accession numbers for mitochondrial and nuclear gene. (DOCX 24 kb)
Additional file 4: STRs observed in *KIAA2018* gene (664 bp) included a proline repeat (CCT motif). The number of repeats ranged 4 to 9 (12 bp to 27 bp). (DOCX 12 kb)

## Abbreviations

AICc: Akaike information criterion corrected
AMOVA: Analysis of Molecular Variance
*APOBE28*: Apolipoprotein B gene exon 28
BAPS: Bayesian Analysis of Population Structure
BEAST: Bayesian Evolutionary Analysis by Sampling Trees
*BLC9L*: Lipoprotein gene
BPEC: Bayesian Phylogeographical and Ecological Clustering
BPP: Bayesian Phylogenetics and Phylogeography
CR: Control Region
*cytb*: Cytochrome b
G: Gamma distribution
HPD: Highest Posterior Density
INDEL: Insertion-deletion
*KIAA2018*: Transcription factor gene
*KIF24*: Kinesin Family Member 24
LGM: Last Glacial Maximum
*MC1R*: Melanocortin-1 Receptor
MCMC: Markov Chain Monte Carlo
MRCA: Most Recent Common Ancestor
MSC: Messinian Salinity Crisis
mtDNA: mitochondrial DNA
*ND*: Nicotine adenine dinucleotide gene
nDNA: Nuclear DNA
phi: Pairwise Homoplasy Index
*RAG1*: Recombination-Activating Gene 1
rjMCMC: reversible jump Markov chain Monte Carlo
rRNA: Ribosomal RNA
STR: Short Tandem Repeat
tRNA: transfer RNA
