# Supplementary material for: Evolutionary history of Podarcis tiliguerta on Corsica and Sardinia
Source: BMC Evol Biol. 2017 Jan 19;17:27. doi: 10.1186/s12862-016-0860-4 (PMC5248522; doi:10.1186/s12862-016-0860-4)
Supplement: Additional file 1: — Primers sequences used in amplification and sequencing. (DOCX 17 kb) [file 12862_2016_860_MOESM1_ESM.docx]

**Additional file 1**

| **Primer** | **Sequence** | **Reference** |
| --- | --- | --- |
|  |  |  |
| 12s rRNA |  |  |
| L1091 | 5′-AAAAAGCTTCAAACTGGGATTAGATACCCCACTAT-3′ | Kocher *et al*. (1989) |
| H1478 | 5′- TGACTGCAGAGGGTGACGGGCGGTGTGT-3′ |  |
|  |  |  |
| Control region |  |  |
| L15022 | 5′-TACCCTTGCTCATAGCATAACTG-3′ | Terrasa *et al*. (2009) |
| H00292 | 5′-GTCTTGTTGACTGTAATTAACCGATA-3′ |  |
|  |  |  |
| ND1-ND2 |  |  |
| L4178 | 5′-CARCTWATACACYTACTATGAAA-3′ | Macey *et al*. (1998) |
| H4980 | 5′-ATTTTTCGTAGTTGGGTTTGRTT-3′ |  |
|  |  |  |
| Cytochrome b *(ii)* |  |  |
| L14724 | 5′-TGACTTGAARAACAYCGTTG-3′ | Palumbi ([1996](file:///C:\Users\Windows\Desktop\MolEcol\nou\paper.docx#_ENREF_51)) |
| H15175 | 5′- CCCTCAGAATGATATTTGTCCTCA-3′ |  |
| L14143 | 5′-CGTTGTATTCAACTATTAAAAY-3′ | This paper |
|  |  |  |
| Cytochrome b *(iii)* |  |  |
| L15347 | 5′-CATGAAACTGGATCAAACAACCC-3′ | Fu (2000) |
| H15915 | 5′- GTCTTCAGTTTTTGGTTTACAAGAC-3′ |  |
|  |  |  |
| 16S rRNA |  |  |
| 16SL1 F | 5′-CCGTGCAAAGGTAGCATAATCAC-3′ | Carranza *et al*. (1999) |
| 16SH1 R | 5′-CCGGTCTGAACTCAGATCACGT -3′ |  |
|  |  |  |
| *RAG1* |  |  |
| RAG-R1 | 5′-AAAATCTGCCTTCCTGTTATTG-3′ | Mayer & Pavlicev (2007) |
| RAG-fo | 5′-GAAAAGGGCTACATCCTGG-3′ |  |
| RAG-re | 5′-CCAGTTATTGCTTTTACAGTTC-3′ |  |
|  |  |  |
| *MC1R* |  |  |
| MC1R-PF | 5′GGCNGCCATYGTCAANAACCGGAACC-3′ | Buades *et al*. (2013) |
| MC1R-PR | 5′CTCCGRAAGGCRTAAATNATGGGGTCCAC-3′ |  |
|  |  |  |
| *APOBE28* |  |  |
| F1 | 5′-TGCGGGAGGAATAYTTTGA-3′ | Portik *et al*. ([2012](file:///C:\Users\Windows\Desktop\MolEcol\nou\paper.docx#_ENREF_56)) with little modifications |
| R1 | 5′-TCTATTCTRAGCTCTCCTTSRCGAA-3′ |  |
|  |  |  |
| *BLC9L* |  |  |
| F1 | 5′-TGGATCCAKCCATGTTTGCTGGG-3′ | Portik *et al*. (2012) with little modifications |
| R1 | 5’-GGCGTCTGCGGGGACTTGAG-3′ |  |
|  |  |  |
| *KIAA2018* |  |  |
| F1 | 5′-CCCATCCYTACCTATGCAGCCATTA-3′ | Portik *et al*. (2012) with little modifications |
| R1 | 5′-TGCCCAGCCATTTGTGATATGCTYTGA-3′ |  |
|  |  |  |
| *KIF24* |  |  |
| F1 | 5′-AAACGTRTCTCCMAAACGCATCC-3′ | Portik *et al*. (2012) with little modifications |
| R1 | 5′-GGCTGCTGRAAYTGCTGGTG-3′ |  |
|  |  |  |
