# Supplementary material for: Evolutionary history of Podarcis tiliguerta on Corsica and Sardinia
Source: BMC Evol Biol. 2017 Jan 19;17:27. doi: 10.1186/s12862-016-0860-4 (PMC5248522; doi:10.1186/s12862-016-0860-4)
Supplement: Additional file 2: — Podarcis spp analysed as outgroup in *BEAST analyses. (DOCX 11 kb) [file 12862_2016_860_MOESM2_ESM.docx]

**Additional file 2**

| **Species** | **Sampled Localities** | **GenBank Accession Numbers*** |
| --- | --- | --- |
|  |  |  |
| ***P. lilfordi*** | Balearic Islands (see Brown *et al*. 2008 for locality details) | **mtDNA:** EF694760–62, EF694764–66, EF694771, EF694773–75, EF694782, EF694785, EF694787–88, EF694799, EU006728, EU006730, EU006734, EU006738, EU006743, EU006745, EU006753, EU006756, EF694797, EF694799, EF694802, EF694805, EF694807, EF694809–10, EF990517, EF990522, EF990525, EF990531, EF990536, EF990540–41, EF990545–46, KC623944 |
| ***P. pityusensis*** | Balearic Islands (see Rodríguez *et al*. 2013 for locality details) | **mtDNA:** EF694768–69, EF694794–95, EU006725–26, EF694814–16, EF990552, EF694827–28, JX852045–JX852108, JX852118–JX852137 |

*Sequences obtained from Brown *et al*. 2008, Rodríguez *et al*. 2013, Terrasa *et al*. 2009 and this paper.
