# Supplementary material for: Evolutionary history of Podarcis tiliguerta on Corsica and Sardinia
Source: BMC Evol Biol. 2017 Jan 19;17:27. doi: 10.1186/s12862-016-0860-4 (PMC5248522; doi:10.1186/s12862-016-0860-4)
Supplement: Additional file 3: — Sample accession numbers for mitochondrial and nuclear gene. (DOCX 24 kb) [file 12862_2016_860_MOESM3_ESM.docx]

| **Specimen** | **12 S rRNA** | **Control Region** | **ND1-ND2** | **Citochrome b** | **16S rRNA** | ***RAG1*** | ***MC1R*** | ***APOBE28*** | ***BLC9L*** | ***KIAA2018*** | ***KIF24*** |
| --- | --- | --- | --- | --- | --- | --- | --- | --- | --- | --- | --- |
| TCO1 | KM58868 | KM58849 | KM58818 | KM58783 | KM58760 | KX237326 | KX237290 | KX237364 | KX237401 | KX237444 | KX237247 |
| TCO2 | KM58869 | KM58850 | KM58819 | KM58784 | KM58761 | KX237327 | KX237291 | KX237365 | KX237402 | KX237445-6 | KX237248 |
| TCO3 | KM58870 | KM58851 | KM58820 | KM58785 | KM58762 | KX237328 | KX237292 | KX237366 | KX237403-4 | KX237447-8 | KX237249 |
| TCO4 | KM58871 | KM58852 | KM58821 | KM58786 | KM58763 | KX237329 | KX237293 | KX237367 | KX237405 | KX237449-50 | KX237250 |
| TCO5 | KM58872 | KM58853 | KM58822 | KM58787 | KM58764 | KX237330 | KX237294 | KX237368 | KX237406 | KX237451-2 | KX237251 |
| TCO6 | KM58872 | KM58854 | KM58823 | KM58788 | KM58764 | KX237331 | KX237295 | KX237369 | KX237407 | KX237453-4 | KX237252-3 |
| TCO7 | KM58873 | KM58855 | KM58824 | KM58789 | KM58765 | KX237332 | KX237296 | KX237370 | KX237408 | KX237455-6 | KX237254 |
| TCO8 | KM58874 | KM58855 | KM58825 | KM58790 | KM58766 | KX237333 | KX237297 | KX237371 | KX237409-10 | KX237457-8 | KX237255 |
| TCO9 | KM58875 | KM58856 | KM58826 | KM58791 | KM58767 | KX237334 | KX237298 | KX237372 | KX237411 | KX237459 | KX237256 |
| TCO10 | KM58876 | KM58857 | KM58827 | KM58792 | KM58767 | KX237335 | KX237299 | KX237373 | KX237412 | KX237460-1 | KX237257-8 |
| TCO11 | KM58877 | KM58858 | KM58828 | KM58793 | KM58768 | KX237336 | KX237300 | KX237374 | KX237413 | KX237462-3 | KX237259 |
| TCO12 | KM58868 | KM58859 | KM58829 | KM58794 | KM58769 | KX237337 | KX237301 | KX237375 | KX237414 | KX237464-5 | KX237260 |
| TCO13 | KM58878 | KM58859 | KM58829 | KM58795 | KM58769 | KX237338 | KX237302 | KX237376 | KX237415 | KX237466-7 | KX237261 |
| TCO14 | KM58868 | KM58859 | KM58829 | KM58796 | KM58782 | KX237339 | KX237303 | KX237377 | KX237416 | KX237468-9 | KX237262 |
| TCO17 | KM58868 | KM58860 | KM58830 | KM58797 | KM58770 | KX237340 | KX237304 | KX237378 | KX237417 | KX237470-1 | KX237263 |
| TCO18 | KM58879 | KM58856 | KM58831 | KM58798 | KM58771 | KX237341 | KX237305 | KX237379 | KX237418 | KX237472 | - |
| TCO19 | KM58877 | KM58861 | KM58828 | KM58799 | KM58768 | KX237342 | KX237306 | KX237380 | KX237419 | KX237473 | - |
| TCO20 | KM58868 | KM58849 | KM58818 | KM58800 | KM58769 | KX237343 | KX237307 | KX237381 | KX237420 | KX237474-5 | KX237264 |
| TCO21 | KM58868 | KM58860 | KM58832 | KM58815 | KM58770 | KX237344 | KX237308 | KX237382 | KX237421 | KX237476-7 | KX237265 |
|  |  |  |  |  |  |  |  |  |  |  |  |
| TSA1 | KM58880 | KM58862 | KM58833 | KM58801 | KM58772 | KX237345 | KX237309 | KX237383 | KX237422 | KX237478-9 | KX237266-7 |
| TSA2 | KM58881 | JX852111 | KM58834 | KM58802 | KM58773 | KX237346 | KX237310 | KX237384 | KX237423-4 | KX237480 | KX237268-9 |
| TSA3 | KM58880 | KM58862 | KM58835 | KM58803 | KM58772 | KX237347 | KX237311 | KX237385 | KX237425-6 | KX237481-2 | KX237270-1 |
| TSA4 | KM58881 | JX852111 | KM58836 | KM58804 | KM58773 | KX237348 | KX237312 | KX237386 | - | KX237483-4 | KX237272 |
| TSA5 | KM58882 | JX852111 | KM58837 | KM58805 | KM58774 | KX237349 | KX237313 | KX237387 | KX237427 | KX237485 | KX237273-4 |
| TSA8 | KM58880 | KM58867 | - | KM58813 | KM58775 | KX237357 | KX237314 | KX237388 | KX237436 | KX237497 | - |
| TSA10 | KM58881 | JX852111 | KM58838 | KM58806 | - | - | - | - | - | - | - |
| TSA11 | KM58883 | KM58863 | KM58839 | KM58807 | - | - | - | - | - | - | - |
| TSA12 | KM58884 | KM58864 | KM58840 | KM58808 | KM58776 | KX237350 | KX237315 | KX237389 | KX237428 | KX237486-7 | KX237275 |
| TSA13 | KM58886 | KM58864 | KM58841 | KM58809 | - | KX237351 | KX237316 | KX237390 | KX237429 | KX237488-9 | KX237276-7 |
| TSA15 | KM58885 | KM58865 | KM58842 | KM58810 | KM58777 | KX237352 | KX237317 | KX237391 | KX237430 | KX237490 | KX237278 |
| TSA17 | KM58881 | JX852111 | KM58834 | KM58802 | - | KX237353 | KX237318 | KX237392 | KX237431-2 | KX237491 | KX237279 |
| TSA18 | KM58884 | KM58864 | KM58843 | KM58811 | KM58776 | KX237354 | KX237319 | KX237393 | KX237433 | KX237492-3 | KX237280 |
| TSA19 | KM58884 | KM58864 | KM58843 | KM58811 | KM58776 | KX237355 | KX237320 | KX237394 | KX237434 | KX237494-5 | - |
| TSA20 | KM58885 | KM58866 | KM58844 | KM58812 | KM58778 | KX237356 | KX237321 | KX237395 | KX237435 | KX237496 | KX237281 |
|  |  |  |  |  |  |  |  |  |  |  |  |
| Tp1 | JX852117 | JX852111 | KM58847 | KM58817 | KM58779 | KX237358 | JX126692 | KX237397 | KX237441 | KX237498 | KX237287 |
| Tp2 | JX852117 | JX852111 | KM58847 | KM58817 | KM58779 | KX237359 | KX237322 | KX237398 | KX237442 | KX237499 | KX237288 |
| Tp3 | JX852117 | JX852111 | KM58847 | KM58817 | KM58779 | KX237360 | KX237323 | KX237399 | KX237443 | KX237500 | - |
| Tf1 | JX852116 | JX852110 | KM58845 | KM58816 | KM58781 | KX237361 | KX237324 | - | KX237437 | KX237502 | KX237282-3 |
| Tf2 | JX852116 | JX852110 | KM58846 | KM58814 | KM58781 | KX237362 | JX126691 | KX237400 | KX237438 | KX237503 | KX237284-5 |
| Tf3 | JX852116 | JX852110 | KM58845 | KM58814 | KM58781 | - | KX237325 | - | KX237439 | KX237504 | KX237286 |
| Ts1 | JX852117 | JX852111 | KM58848 | KM58817 | KM58780 | KX237363 | JX126693 | KX237396 | KX237440 | KX237501 | KX237289 |
