# Supplementary material for: Evolutionary history of Podarcis tiliguerta on Corsica and Sardinia
Source: BMC Evol Biol. 2017 Jan 19;17:27. doi: 10.1186/s12862-016-0860-4 (PMC5248522; doi:10.1186/s12862-016-0860-4)
Supplement: Additional file 4: — STRs observed in KIAA2018 gene (664 bp) included a proline repeat (CCT motif). The number of repeats ranged 4 to 9 (12 bp to 27 bp). (DOCX 12 kb) [file 12862_2016_860_MOESM4_ESM.docx]

**Additional file 4.**

| Sample | Genotype | Sample | Genotype |
| --- | --- | --- | --- |
|  | | | |
| **Sardinia Island** | | **Corsica Island** | |
|  | |  | |
| TSA1 | 6-9 | TCO1 | 6-6 |
| TSA2 | 6-6 | TCO2 | 7-8 |
| TSA3 | 7-9 | TCO3 | 6-8 |
| TSA4 | 7-9 | TCO4 | 6-9 |
| TSA5 | 7-7 | TCO5 | 4-7 |
| TSA12 | 6-9 | TCO6 | 6-7 |
| TSA13 | 6-9 | TCO7 | 5-7 |
| TSA15 | 6-6 | TCO8 | 5-8 |
| TSA17 | 7-7 | TCO9 | 8-8 |
| TSA18 | 7-9 | TCO10 | 7-9 |
| TSA19 | 6-9 | TCO11 | 7-8 |
| TSA20 | 6-6 | TCO12 | 5-6 |
|  |  | TCO13 | 6-7 |
| **Sardinian islands and islets** | | TCO14 | 5-7 |
|  |  | TCO17 | 5-7 |
| TSA8 | 9-9 | TCO18 | 7-7 |
| Tp1 | 7-7 | TCO19 | 7-7 |
| Tp2 | 7-7 | TCO20 | 6-8 |
| Tp3 | 7-7 | TCO21 | 4-8 |
| Ts1 | 8-8 |  |  |
| Tf1 | 8-8 |  |  |
| Tf2 | 9-9 |  |  |
| Tf3 | 8-8 |  |  |
|  |  |  |  |
